# Awareness of Child Abuse and Neglect Among the General Public in Saudi Arabia: A Systematic Review

**DOI:** 10.7759/cureus.32550

**Published:** 2022-12-15

**Authors:** Mohammad Hussen Sheikh, Abeer Mohammed M Alanazi, Dina Ahmed Aljohani, Lama Mueysh M Aljohani, Manal Mohammed S Alatawi, Ahlam Shary J Hazazi, Amal Abutaleb M Qaysi, Dhuha Abdullah H ALQasir, Sarah Awad M Alenzi, Ibtisam Shary J Hazazi, Alhanoof Abdulhakeem Hazazi, Shouq Abdullah O Alwabisi, Renad Mohammed H Alanazi, Alaa Ayoub Baqadu

**Affiliations:** 1 Pediatrics Department, Faculty of Medicine, University of Tabuk, Tabuk, SAU; 2 Pediatrics Department, Faculty of Medicine, Kasr Al-Ainy School of Medicine, Cairo University, Cairo, EGY; 3 Pediatrics Department, Faculty of Medicine, Almaarefa University, Riyadh, SAU

**Keywords:** general public, saudi arabia, child abuse, beliefs, awareness

## Abstract

Child abuse represents a serious problem worldwide. In Arab countries, the problem is complicated because abuse may be perceived as a method of discipline. This review aimed to describe awareness of the public in Saudi Arabia regarding child abuse. A search was performed in the databases of MEDLINE/PubMed, Scopus, Academic Search Complete (EBSCOhost), and Web of Science for articles published in English from the 1^st^ of January, 2000, to the 14^th^ of November, 2022. The search was conducted during the period from the 7^th^ to the 14^th^ of November 2022. The used search words were {“Child Abuse”} AND {"Saudi Arabia"} AND {“awareness”}. The risk of bias (ROB) was assessed using the Risk of Bias Instrument for Cross-Sectional Surveys of Attitudes and Practices. Six studies were included in this review. Most studies had high ROB in recruiting the participants, designing the questionnaires, and stating the rate of response. The awareness regarding physical abuse seemed fair in most studies, but the awareness about shaken baby syndrome was poor. Also, there was a misconception about the parent's right to discipline their children through corporal punishment. Most participants did not perceive a need for establishing protective laws or programs. Public awareness about emotional abuse and neglect was lower than in cases of physical abuse. The overall knowledge about child abuse seems to be fair, but poor knowledge was observed in some forms, such as shaken baby syndrome. The public concepts about physical punishment and the need for protective laws and programs are also negative and require more efforts to alter them.

## Introduction and background

Child abuse is a worldwide serious problem that can lead to life-long consequences and even mortality. Child maltreatment is defined as “all forms of physical and emotional ill-treatment, sexual abuse, neglect, and exploitation that results in actual or potential harm to the child’s health, development, or dignity” [1]. According to the World Health Organization, 300 million children between the age of two and four years suffer from corporal punishment and/or psychological maltreatment that are inflicted by their parents and caregivers [2].

Child abuse can present in different forms as physical abuse, psychological/ emotional abuse, sexual abuse, and neglect. The abuse may occur through an act of commission (such as hitting or humiliation by words) or an act of omission (e.g., not providing the child with essential needs such as food or proper clothing for the weather) [3].

Several consequences can result from child abuse and can last for the life of the victim or cause death. These consequences may be physical and/or psychological. Physical consequences comprise neurologic deficits, developmental delays, cerebral palsy, or other forms of disability according to the nature and the site of the inflicted injuries. Psychological consequences include a higher risk of suffering depression and conduct disorder, as well as becoming dependent on substances. Abused children may also exhibit poor academic performance at school due to decreased cognitive function [1].

Studies from Saudi Arabia reported various forms of child abuse [4-7]. To reduce the rate of child abuse, the Saudi government initiated the National Family Safety Program (NFSP) in 2005, which became under the administration of King Abdul Aziz Medical City [8]. During the COVID-19 pandemic, the reporting of child maltreatment cases to the NFSP in Saudi Arabia showed some changes compared to the pre-COVID-19 era. For example, a significantly higher rate of abuse cases was reported by a family member than by the victims or by a healthcare worker. The victims during the pandemic were older than the pre-pandemic cases [9]. The reported types of abuse also differed, with higher rates of emotional and sexual abuse during the COVID-19 epidemic compared to pre-COVID rates [9,10]. Children had a higher probability by 1.69 times to suffer sexual abuse during the pandemic compared to the period before the pandemic. Also, the COVID-19 pandemic was significantly associated with a lower probability of physical child abuse (47.7% less) [10].

As prevention is better than cure, an essential step to reduce the suffering of abused children is to increase public awareness about child maltreatment. When public awareness increases about this problem, abusive practices will become condemned by society, and the rate of abuse will decrease. In addition, a society with increased awareness can take active measures, such as reporting the abuse to the concerned authorities and aiding the child in getting help and support.

Assessing the awareness of the public can help also in designing future awareness campaigns to address the misconceptions held by the public so that the public will recognize the abusive practices that they now consider normal and harmless and will hopefully cease to perform them in the future.

Some studies were conducted to assess the awareness of parents, teachers, health professionals, and the public about child abuse [11-13]. However, most of these studies focused on health professionals and teachers who are assumed to have more knowledge due to their training. There is a need to summarize the conceptions of the public about this serious problem to guide future preventive programs.

Therefore, this systematic review aimed to describe the public awareness in Saudi Arabia regarding child abuse and to summarize the factors influencing awareness.

## Review

Methods

Methodology

This systematic review was conducted according to the principles of the Cochrane Handbook for Systematic Reviews of Interventions, version 6. The reporting followed the Preferred Reporting Items for Systematic Reviews and Meta-Analyses (PRISMA) guidelines [14].

The Research Question

Does the public in Saudi Arabia have sufficient awareness regarding child abuse?

Research Aim and Objectives

This systematic review aimed at investigating the awareness of the public in Saudi Arabia about child abuse. The following objectives were addressed: (a) to assess the level of awareness of the public towards the different forms of abuse (physical, sexual, emotional, and neglect) and (b) to identify the factors influencing the level of awareness.

Inclusion Criteria for Studies

This systematic review included cross-sectional survey studies that were published in English during the period from the 1^st^ of January 2000 to the 14^th^ of November 2022. We did not include studies before 2000 as it was expected that a marked difference in awareness level will be observed between older and more recent studies, owing to the enormous efforts for enhancing awareness that the Saudi government exerted through NFSP.

The included studies were conducted on adult individuals of the public in Saudi Arabia. Studies that included questionnaires assessing awareness, perception, or beliefs about any form of child abuse were included. We excluded types of publications other than surveys (case reports, case series, review articles, editorials, commentaries), as well as conference abstracts/posters. We also excluded surveys that investigated the prevalence rates or risk factors of abuse rather than the awareness or knowledge about child abuse. The review excluded studies that addressed the awareness of professionals only as healthcare workers, medical/nursing/dental students, or teachers. These categories are expected to have necessarily higher levels of awareness owing to the inclusion of the subject of child abuse in their education and training.

Search Strategy

We searched the electronic databases of MEDLINE/PubMed, Scopus, Academic Search Complete (EBSCOhost), and Web of Science for studies published in English during the period from the 1^st^ of January 2000 to the 14^th^ of November 2022. The search process was carried out between November 1, 2022, and November 14, 2022. The terms used for the search were {“Child Abuse”} AND {"Saudi Arabia"} AND {“awareness”}. The reference lists of studies that were obtained by the electronic search were scrutinized to identify relevant studies.

Selection of Studies

ISJH, SAOA, and RMHA conducted the search process. The yielded results were screened through their titles and abstracts to remove duplicates as well as the types of publications to be excluded and non-relevant studies. For the identified potentially relevant articles, their full text was retrieved and assessed for eligibility. We checked the search results as well the selection process and the causes for the exclusion of studies.

Data Extraction

AMMA, MMSA, AAH, SAMA, and DAHA extracted the data from the included studies into a standardized data extraction sheet. The extracted data included: (a) the study characteristics (the study duration, sampling method, number of participants, response rate, and focus of the questionnaire); (b) the respondents’ characteristics (the age, sex, educational status, and having children or not); and (c) the questionnaire used: open/closed questions, the methods of devising and distributing the questionnaire, response rate, and the focus of the questions (physical, sexual, or emotional abuse or neglect). We revised the collected data regarding their consistency and clarity.

Measured Outcomes

The primary outcomes included the level of awareness of the participants regarding child abuse and the most frequent areas of defective knowledge or misconceptions. The secondary outcomes included the factors that affect the awareness level of the public.

Assessment of the Risk of Bias in the Included Studies

AMMA, MMSA, AAH, SAMA, and DAHA assessed the risk of bias (ROB) for the included studies using the “Risk of Bias Instrument for Cross-Sectional Surveys of Attitudes and Practices” developed by the CLARITY Group at McMaster University in conjunction with Evidence Partners. The tool consists of five questions, and each is given a score of “definitely yes” (low ROB), “probably yes”, “probably no” (higher ROB), and “definitely no” (high ROB). The questions assess whether the source population is representative of the population of interest, the adequacy of the response rate, the amount of missing data, the clinical sensibility of the survey, and the reliability and validity of the survey instrument.

Results

Results of Literature Search and Study Selection

**Figure 1:**
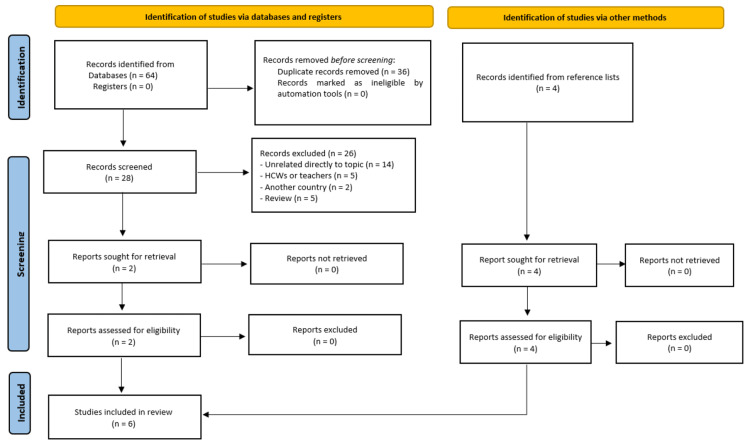
The PRISMA flow chart diagram for the results of the search and study selection. n: Number; HCWs: Healthcare workers; PRISMA: Preferred reporting items for systematic reviews and meta-analyses

The PRISMA 2020 flowchart that summarizes the results of the literature search and study selection can be seen in Figure 1.

The search of the electronic databases yielded 64 records from databases. After removing the duplicates, 28 records were screened for the titles and abstracts, out of which 26 records were non-relevant to the research question or the type of publication and were excluded. The full text of the remaining two studies was examined for eligibility [15,16]. In addition, four more studies [17-20] were identified through the screening of the reference lists of the articles and fulfilled the eligibility criteria. Finally, six studies were included in this systematic review [15-20].

Basic Characteristics of the Included Studies

Four studies were conducted in Riyadh, which is the capital of Saudi Arabia [15,17-19], while one study included participants from all over Saudi Arabia [20], and the location of one study was not stated. The studied population was the parents of children in four studies [15-18], while two studies addressed the adult public [19,20]. Alanazi [17] collected the participants from various sites, such as workplaces (e.g., ministries, hospitals, schools, and companies) as well as training institutions and visitors to Riyadh employment and labor offices. The participants were selected from three primary healthcare centers in the study by Al Dosari et al. [18] but were invited through social media platforms in the study by AlOmran et al. [15]. The other three studies did not mention how the participants were recruited [16,19,20]. The sample size varied greatly from 200 up to 4,117 participants, and sample size calculations were explained in two studies only [15,18]. One study [18] clearly stated the random selection of the participants, while the remaining studies either did not mention or explain random selection (Table 1).

**Table 1:** Characteristics of the included studies and the studied populations and samples (total n = 6).

Study	Region	Time Period	Population	Place of Collecting Sample	Calculated Sample Size	Sample Size	Random Selection
Alanazi [17]	Riyadh	Feb 2007 to Apr 2007	Parents of Children + young adults	Several sites	530	530	No
Al Dosari et al. [18]	Riyadh	Oct 2009 to Mar 2010	Parents of Children	Parents of children attending three primary health care centres	200	200	Yes
Alqurashi et al. [19]	Riyadh	May 2018 to Apr 2019	Adult General Population	Not clear	Not done	4,117	No
Alreshidi et al. [16]	Not clear	Feb 2019 to Feb 2020.	Parents of Children	Not clear	Not done	400	Wrote random but method not described
Alreshidi et al. [20]	All over Saudi Arabia	Not stated	Adult General Population	Not clear	Not explained	454	Not clear
AlOmran et al. [15]	Riyadh	Jan 2021 to Sep2021	Parents of Children	Distributed on social media platforms	385	577	No

As regards the focus of the included studies, different types of abuse were assessed in the studies by Alqurashi et al. [19] and Alreshidi et al. [20]. Alanazi [17] and Al Dosari et al. [18] addressed physical abuse, whereas the focus was on the shaken baby syndrome in the study by AlOmran et al. [15] and neglect in the study by Alreshidi et al. [16]. Alanazi [17] devised a new questionnaire based on previous studies. Al Dosari et al. [18] based their questionnaire on two previously validated questionnaires, while the remaining studies did not mention how their questionnaires were devised. The questionnaire was pretested only in the studies by Alanazi [17] and Alreshidi et al. [20]. Alanazi [17] and Al Dosari et al. [18] distributed the questionnaires as self-administered printed forms, but Alreshidi et al. [16] conducted personal interviews with the participants. The forms were electronic online in the studies by Alreshidi et al. [20] and AlOmran et al. [15]. Alqurashi et al. [19] did not clearly state the method of distribution of the questionnaire. The response rate was mentioned only in the study of Al Dosari et al. [18] and was 91% (Table 2).

**Table 2:** Characteristics of the used questionnaires in the included studies (total n = 6).

First author, year of publication	Focus	Tools	Method of Distributing Questionnaires	Response Rate
Alanazi [17]	Physical abuse	Devised questionnaire whose basis was explained	Self-administered, printed forms	63.1%
Al Dosari et al. [18]	Physical abuse	Arabic language self-administered questionnaire based on “Childhood Trauma Questionnaire” & “Parenting Profile Assessment”	Self-administered, printed forms	91 %
Alqurashi et al. [19]	Different types of abuse	Devised questionnaire whose basis was not stated	Not clear	Not stated
Alreshidi et al. [16]	Neglect	Devised questionnaire but the basis was not stated	Personal interview	Not stated
Alreshidi et al. [20]	Different types of abuse	Pretested questionnaire but the basis is not mentioned	Self-administered, online questionnaire	Not stated
AlOmran et al. [15]	Shaken baby syndrome	Devised questionnaire whose basis was not stated	Validated, electronic questionnaires	Not stated

Summary of the Included Studies

Alanazi [17] distributed 840 questionnaires, and 530 were returned and included in the analysis. Among the 530 participants, 53.8% were parents, and 46.2% were young people. Most parents (86%) used physical punishment on their children, while only 14% of parents never punished any of their children at any time. Most parents agreed or strongly agreed that parents need to use physical punishment as a disciplinary method (81 %), that physical punishment is a useful disciplinary method (61%), and that physical punishment is an acceptable action by parents (62%). Meanwhile, 60% of parents believed that physical punishment is harmful to children. Most parents (58%) disagreed or strongly disagreed with the legislative prevention of the parental use of physical punishment. On the other hand, most parents (74%) supported the issuing of laws to prevent parents from using severe physical punishment. The attitudes of young people towards physical punishment widely ranged. Half of the young people agreed that parents have a right to discipline their children and that physical punishment is a useful method of discipline. However, 58% of young people stated that physical punishment needs to be prevented by the law.

In their study, Al Dosari et al. [18] recruited 200 parents who were attending three primary healthcare centres in Riyadh. They used a self-administered, printed questionnaire form. The questionnaire covered five main risk domains (personal factors, history of parents’ childhood abuse, parental attitude toward punishment, family effects and community factors). They found that 34% of the respondents had a history of physical abuse in their childhood. Physical punishment was used by 18% of parents. Parents reported some beliefs that indicate inadequate awareness about abuse, including accepting hitting children (59.9%), non-considering hitting as a form of child abuse (81%), the effectiveness of hitting in noisy children (37.9%), the effectiveness of hitting children as an educational tool (61.5%), the right of the parents to discipline their children (70.9%), difficulty in differentiating between physical punishment and child abuse (62.9%), the parental right to discipline one’s child, and the lack of need for a law to prevent child abuse (73.3%) or to punish abusive parents (81.8%).

Alqurashi et al. [19] aimed to assess the awareness of the public regarding the child’s rights. They distributed a questionnaire among the adult public in Riyadh City, though the details of distributing the questionnaire (e.g., printed or electronic, the method of recruiting the sample) were not clearly explained. The questionnaire inquired about the respondents’ socio-demographic data as well as their awareness and knowledge about child abuse. The respondents displayed a good knowledge level about the neglect of physical and educational needs as well as physical abuse. The respondents’ answers stated that emotional and physical forms of abuse were the most frequent types of abuse in the community. Most respondents did not know where to seek help for reporting child abuse cases. The defect areas observed in the respondents' answers were “Locking the child alone at home for many hours” and “Cursing the day the child was born” where only 45.4% and 57.2%, respectively, identified these as emotional abuse. “Burning a child who wets his/her bed while sleeping” was identified as physical abuse by 49.1% only. “Reluctance to monitor the child’s growth periodically” and “Late consultation of medical care when the child is ill” were recognized as neglect by 53.7% and 47.9%, respectively. The mean score knowledge was lowest for educational neglect, followed by physical neglect, and then emotional abuse. The highest mean knowledge score was physical abuse and medical care neglect. Good knowledge was significantly associated with the male gender, education (Bachelor or postgraduate degrees), and the occupation of the parents (professional or military father jobs and mothers' professional job). No association was observed with age, marital status, or the number of children in the family.

Alreshidi et al. [16] assessed the perceptions of Saudi parents towards factors related to child abuse and neglect. The researchers enrolled 400 Saudi parents who were personally interviewed. The questionnaire included questions about the parents’ demographic data and questions assessing awareness about child neglect (e.g., going to schools, taking treatment of child cleanliness, and child playing). They found that higher parents’ awareness was significantly associated with the male gender. This study had serious limitations, including the non-clarity of the distribution process of the questionnaire and how the sample was selected. Also, statistical tests and comparisons were not clear to derive sound conclusions.

Alreshidi et al. [20] explored the perspectives of the adult public about different types of child abuse. The investigators collected data using a pretested, self-administered online survey. The study included 454 respondents. The most reported type of abuse by the respondents was emotional abuse and neglect. Most (161/454, 35.5%) of the respondents thought that the best approach is family education over child abuse, while only 67 (14.8%) believed that rehabilitation is the best method of protecting the child against abuse.

AlOmran et al. [15] investigated the awareness, knowledge level, and attitude of the parents of children - in Riyadh, Saudi Arabia - regarding shaken baby syndrome and identified the factors affecting their knowledge. The investigators used a validated electronic questionnaire, mentioning that the random sampling method was used though not explaining its details. The study recruited 577 respondents. Only one-third (32.1%) admitted hearing before about the shaken baby syndrome. They found that the respondents knowledge was poor in 81.3% of parents; however, most parents had a positive attitude (82.5%). One-quarter of participants thought that shaking a child violently produces no consequences, and 71.2% believed that shaking cannot lead to death. Yet, most respondents (89.4%) agreed that the shaken baby syndrome can be prevented. The sources of the respondents’ knowledge were mostly the internet and social media (42.2%), with less frequency of medical or health authorities’ resources. Good knowledge level was significantly associated with the female gender, being widowed (compared to married/divorced), and being a student (compared to housewife/employee). No significant association was observed with the age of the parent, educational level, or the number of children they had.

Assessment of the Risk of Bias in the Included Studies

The ROB of the included studies was assessed using the “Risk of Bias Instrument for Cross-Sectional Surveys of Attitudes and Practices” developed by the CLARITY Group at McMaster University in conjunction with Evidence Partners (Table 3).

**Table 3:** Assessment of the risk of bias of the included studies (total n = 6). ROB: Risk of bias

First author, year of publication	1. Is the source population representative of the population of interest?	2. Is the response rate adequate?	3. Is there little missing data?	4. Is the survey clinically sensible?	5. Is there any evidence for the reliability and validity of the survey instrument?
Alanazi [17]	Probably yes	Probably yes	Definitely yes (low ROB)	Definitely yes (low ROB)	Definitely no (high ROB)
Al Dosari et al. [18]	Definitely yes (low ROB)	Definitely yes (low ROB)	Definitely yes (low ROB)	Probably yes	Probably yes
Alqurashi et al. [19]	Probably no	Definitely no (high ROB)	Definitely yes (low ROB)	Definitely no (high ROB)	Definitely no (high ROB)
Alreshidi et al. [16]	Probably no	Definitely no (high ROB)	Definitely yes (low ROB)	Definitely no (high ROB)	Definitely no (high ROB)
Alreshidi et al. [20]	Probably yes	Definitely no (high ROB)	Definitely yes (low ROB)	Definitely no (high ROB)	Definitely no (high ROB)
AlOmran et al. [15]	Probably no	Definitely no (high ROB)	Definitely yes (low ROB)	Definitely yes (low ROB)	Definitely no (high ROB)

The source of the population was representative of the population of interest only in the study by Al Dosari et al. [18], who recruited the participants from three primary healthcare centers and used the random sampling technique (low ROB). Alanazi [17] recruited the participants from various sites, but the sampling method was purposive (higher ROB). The remaining four studies derived their participants from a single center only or did not give enough information about the place or the sampling method (higher ROB).

As regards the response rate, Al Dosari et al. [18] was the only study that provided an adequate response rate (low ROB). The response rate was much lower in the study by Alanazi [17] (higher ROB). The other studies neither provided information about the response rate nor mentioned the number of distributed questionnaires to enable the calculation of response rates (high ROB). Examination of the tables of the included studies showed either a lack of missing data or a small percentage not exceeding 10% (low ROB).

The clinical sensibility of the survey referred to “the formal assessment of comprehensiveness, clarity, and face validity of the questionnaire in a similar population.” The studies by Alanazi [17] and AlOmran et al. [15] only described pretesting of the questionnaire on a pilot sample (low ROB). Al Dosari et al. [18] utilized questionnaires that were validated in populations different from theirs, so the ROB was slightly higher. The other three studies [16,19,20] neither used a validated questionnaire form nor performed pretesting on a pilot sample (high ROB).

The evidence of reliability and validity was available only for the questionnaire in the study by Al Dosari et al. [18], though these were derived from other different populations (slightly higher ROB). The remaining five studies did not provide data on the validity and reliability of the questionnaires used [15-17,19,20]. The details of the questionnaire development were obscure in four studies, with the basis for the choice of questions not explained [15,16,19,20] (high ROB).

Discussion

Summary of Main Results

This systematic review was undertaken to describe the awareness of the public in Saudi Arabia regarding child abuse and to summarize the factors influencing awareness. Six studies were retrieved that fulfilled the eligibility criteria set for this review. The studies showed considerable heterogeneity in the included questions, so pooling of the data was not feasible.

As regards awareness about physical abuse, five studies were found [15,17-20]. Alanazi [17] and Al Dosari et al. [18] assessed solely the physical abuse, and AlOmran et al. [15] focused only on the shaken baby syndrome. The studies by Alqurashi et al. [19] and Alreshidi et al. [20] assessed physical abuse along with other types of abuse. The public or parents' knowledge about what constitutes physical abuse was fair in three studies [18-20]. However, a considerable and alarming percentage of parents in the studies by Alanazi [17] in 2008 and Al Dosari et al. [18] in 2017 held misconceptions about the right of parents to hit their children as an effective method for dealing with noisy children or as an educational or disciplinary method. This finding was echoed to some extent in the study by Alqurashi et al. [19], who found that burning a child who wets the bed while sleeping was not recognized as abuse by more than half the participants. In addition, Alanazi [17] and Al Dosari et al. [18] found that most parents did not perceive a need for a law to prevent abuse or to punish abusive parents, implying that parents’ misconceptions can oppose the efforts exerted by the Saudi government and concerned organizations towards improving the reporting of child abuse and reducing its prevalence.

Previous studies in Saudi Arabia regarding the barriers to reporting child abuse have identified these misconceptions about the parents' rights in hitting their children, which stems from a mistaken interpretation of Islamic teachings. Interestingly, slapping a child was condemned as abuse by most parents in the studies by Al Dosari et al. [18], Alqurashi et al. [19], and Alreshidi et al. [20], but hitting the child with a stick or slipper was less recognized as abuse. This disparity could also be explained considering the understanding of the public of Islamic teachings, as slapping on the face is prohibited by the Prophet. Overcoming this perspective of the child abuse program requires the incorporation of the efforts of religious figures to explain the difference between abuse and discipline and to emphasize the rights of children in Islam [21].

Shaking of the children was the principal issue in the study by AlOmran et al. [15], and it was also addressed in a single question in the study by Alreshidi et al. [20]. Alreshidi et al. [20] found that the awareness of the public that violent shaking constitutes abuse was moderate, while AlOmran et al. [15] reported a poor awareness of the parents that shaking is a form of abuse that can cause serious health issues and death. These results draw attention that some forms of child abuse, such as violent shaking, might not be highlighted enough in the awareness campaigns and require more illustration.

Awareness about sexual child abuse was assessed by Alreshidi et al. [20]. They found that the public had moderate to good knowledge regarding what constitutes sexual abuse of the child, such as appearing naked in front of the child, playing with the child’s private parts, and having sex with the child. Alqurashi et al. [19] asked the parents about the effective methods that can protect their children from being victims of sexual abuse. The percentage of parents who selected open communication with their children was slightly above half the respondents, reflecting that the parents might find it embarrassing to have open communication with their children about this issue. This finding suggests the inclusion of methods to prevent sexual child abuse within the awareness campaigns and teaching the parents’ how to communicate with their children and how to explain such a sensitive issue.

Awareness about emotional abuse was assessed by Alqurashi et al. [19] and Alreshidi et al. [20]. The respondents' awareness about practices that constitute emotional abuse was fair and tended to be less than their awareness of physical or sexual abuse, except in the extreme practices of emotional abuse as locking a child inside or outside the house. Respondents in the two studies regarded emotional abuse as the most prevalent type of child abuse. This may be attributed to the lack of visible damage by emotional abuse as opposed to physical and sexual abuse, which causes injuries and even infirmities. Emotional abuse can lead to serious psychological consequences that are not immediately seen by abusive parents or caregivers [1]. Awareness should be raised regarding the damaging effect of emotional abuse in the long run and that the resultant psychological trauma is comparable to the injuries and scars left by physical abuse or may even be more serious.

As regards awareness about child neglect, three studies were found [16,19,20]. Alqurashi et al. [19] asked about different forms of neglect (physical, safety, medical care, and educational). They found that awareness about medical care neglect was good, but awareness about safety neglect was moderate. Awareness about physical and educational neglect was rather poor. Alreshidi et al. [20] did not thoroughly investigate the forms of neglect as Alqurashi et al. [19], asking only about ignoring the signs of illness, caring about the child’s food, and monitoring the child’s interaction with the internet. The responses to these questions showed moderate to good awareness. The study by Alreshidi et al. [16] focused on neglect and asked about several aspects as cleanliness, playing, administering treatment, and education. The least recognized forms of neglect were prohibiting playing and administering treatment. Neglect was called by a previous study “the neglected form of child maltreatment” [7], as it is less recognized than the other forms of abuse in which damage is produced through commission.

The factors that affect public awareness about child abuse were investigated in some studies [15,16,19], with controversial results. Male gender was significantly associated with good knowledge, as reported by Alqurashi et al. [19] and Alreshidi et al. [16], while females had better knowledge in the study by AlOmran et al. [15]. Higher education and professional jobs were significantly associated with good knowledge in the study by Alqurashi et al. [19] but not in the other two studies [15,16]. This controversy could be attributed to differences in the used questions to assess knowledge. None of these studies conducted a multivariate analysis to adjust for the effects of confounders, such as the age or having a professional education/training about child abuse, as is the case with healthcare workers and teachers.

Overall Completeness, Applicability, and Quality of the Evidence

The included studies showed a high ROB in several domains, which could impact the results and the derived implications. There is a need to initiate studies to assess public awareness and adhere to the best methodological quality measures as using the random sampling technique, recruiting respondents from multiple centers or areas, using validated questionnaires, and reporting the response rate. Noticeably, four out of the included six studies were conducted in Riyadh, the capital of Saudi Arabia [15,17-19]. Only one study included respondents from all over Saudi Arabia [20]. This factor can also limit the generalizability of the results, as awareness in capital cities is expected to be higher than in rural areas. Future studies should consider the recruitment of respondents from various regions of Saudi Arabia to represent the whole cultural and educational spectrum of Saudi society.

Agreements and Disagreements With Other Studies or Reviews

To the best of the authors’ knowledge, no previous systematic reviews assessed public awareness about child abuse. However, some of the discussed concepts in the included studies were mentioned in narrative reviews that discussed barriers against reporting child abuse in Saudi Arabia [22], such as the misconceptions about the parents’ rights in hitting their children and that hitting is permitted in Islamic teachings as a disciplinary measure.

Importance of Awareness Campaigns

Awareness campaigns have demonstrated their effectiveness in elevating the level of knowledge of the public. In Saudi Arabia, Temsah et al. [23] conducted a pre-post- experimental study on 308 parents who attended a childhood and adolescence safety campaign. They found that the parents’ knowledge significantly improved from 36.2 ± 17.7 to 79.3 ± 15.6 after attending the campaign. Another example is the Florida Winds of Change campaign, which showed a significant improvement in the participants’ knowledge of child development and the available community resources for parents. In addition, the participants’ attitudes became more positive toward the prevention of child abuse and neglect [24].

## Conclusions

The results of this systematic review provide insights into the level of public awareness about child abuse. The public awareness was fair to good, particularly in the cases of physical and sexual abuse, but the practices of emotional abuse and neglect were less recognized. Also, the shaken baby syndrome was not known to the public as a form of abuse, and the potential of developing serious injuries was not perceived by most respondents. This warrants the designing of awareness campaigns that emphasize these neglected aspects of child abuse to improve public awareness about them.

The misconceptions held by the public that were summarized in this review should be overcome to ensure the cooperation of the public with the established programs and to create a sound culture of child’s rights and safety in Saudi society. In addition, the results of this review can guide future research in this area as the review highlighted the pitfalls and limitations in the currently available studies.
